# Retinal Capillary Rarefaction in Patients with Type 2 Diabetes Mellitus

**DOI:** 10.1371/journal.pone.0162608

**Published:** 2016-12-09

**Authors:** Agnes Jumar, Joanna M. Harazny, Christian Ott, Stefanie Friedrich, Iris Kistner, Kristina Striepe, Roland E. Schmieder

**Affiliations:** 1 Department of Nephrology and Hypertension, Friedrich-Alexander-University Erlangen-Nürnberg (FAU), Germany; 2 Department of Pathophysiology, University of Warmia and Mazury Olsztyn, Poland; International University of Health and Welfare, JAPAN

## Abstract

**Purpose:**

In diabetes mellitus type 2, capillary rarefaction plays a pivotal role in the pathogenesis of end-organ damage. We investigated retinal capillary density in patients with early disease.

**Methods:**

This cross-sectional study compares retinal capillary rarefaction determined by intercapillary distance (ICD) and capillary area (CapA), measured non-invasively and in vivo by scanning laser Doppler flowmetry, in 73 patients with type 2 diabetes, 55 healthy controls and 134 individuals with hypertension stage 1 or 2.

**Results:**

In diabetic patients, ICD was greater (23.2±5.5 vs 20.2±4.2, p = 0.013) and CapA smaller (1592±595 vs 1821±652, p = 0.019) than in healthy controls after adjustment for differences in cardiovascular risk factors between the groups. Compared to hypertensive patients, diabetic individuals showed no difference in ICD (23.1±5.8, p = 0.781) and CapA (1556±649, p = 0.768).

**Conclusion:**

In the early stage of diabetes type 2, patients showed capillary rarefaction compared to healthy individuals.

## Introduction

Diabetes mellitus, a major risk factor for cardiovascular disease, alters microvascular structure and function. High glucose concentration has been shown to cause endothelial cell dysfunction, for example mediated by vasoconstrictor prostanoids[1], and impaires nitric oxide activity.[2],[3] Nitric oxide seems to be involved in vascular endothelial growth factor-induced angiogenesis.[4] Evidence from animal models supports the pathophysiological concept of diabetes-induced impairment of angiogenesis and collateral vessel formation.

Microvascular rarefaction is described as result of induction of specialized antiangiogenic programs predominantly mediated by angiostatin and endostatin[5]. Furthermore induction of diabetes type 1 induced a decrease in major angiogenic growth factor in the skeletal muscle capillary bed of mice.[6] In a chicken model of neoangiogenesis, hyperglycemia was shown to impair angiogenesis through induction of apoptosis and decreases proliferation of endothelial cells.[7] In a diabetic mouse model with hindlimb-induced ischemia, restoration of perfusion in the ischemic limb was impaired and capillary density reduced.[8] Goligorsky and colleagues demonstrated an angiogenic switch during natural history of type 2 diabetes mellitus (T2DM) from pro-angiogenic phenotype at the early pre-diabetic stages to the profound inhibition of ex-vivo angiogenesis and microvascular rarefaction in the kidneys of obese zucker diabetic fat rats.[5] Peritubular capillary rarefaction is a major hallmark of chronic kidney disease and predicts renal outcome in diabetic nephropathy.[9] There is evidence from animal models that endothelial dysfunction, oxidative stress, interstitial hypoxia due to arteriolar vasoconstriction and pericyte detachment from the vasculature are involved in this pathophysiological process.[9]

Data about capillary density In patients with T2DM and overt end-organ damage are heterogeneous. In patients with diabetes mellitus (mixed population of type 1 and 2) and advanced stages of coronary atherosclerosis, decreased coronary collateral vessel formation was found compared to non-diabetic patients.[3] Capillary videomicroscopy of dorsal finger skin of patients with T2DM (50% of the patients presented with either diabetic retinopathy or microalbuminuria) showed no difference in capillary density compared to healthy controls.[10] In conjunctival vessels of patients with diabetes mellitus (mixed population of type 1 and 2), fewer capillaries and postcapillaries were found in comparison with non-diabetic patients.[11] Human in-vivo data about capillary rarefaction at the early stage of diabetes mellitus type 2 without overt end-organ damage is missing.

Capillary rarefaction is increasingly believed to play a pivotal role in the pathogenesis of end-organ damage by affecting pressure and blood flow pattern. This severely affects peripheral vascular resistance (and thereby on systemic blood pressure), metabolisms[12, 13] and notably promotes tissue ischemia.[14–16] The retinal circulation allows an inside into the microcirculation in general[17] and is believed to mirror the cerebrovascular circulation in particular.[18–20] Vast knowledge exists about diabetic retinopathy, a well-known example of overt end-organ damage[21]. However, although allowing for the detection of very early changes in retinal capillary density, assessment of retinal capillary density with scanning laser Doppler flowmetry (SLDF)^[^^22^^]^ has not been described in patients with T2DM yet. SLDF of retinal vessels allows for the investigation of capillary area (CapA), and intercapillary distance (ICD), both reflecting capillary density. T2DM often coexists with hypertension. The objective of this study was to compare retinal capillary density in patients with early stage of T2DM, healthy controls and hypertensive patients.

## Methods

The study population comprised patients who participated in one of four randomized, double blind, mono-center clinical trials during November 2005 and April 2015 in our Clinical Research Unit at the Department of Nephrology and Hypertension, University of Erlangen-Nürnberg, Germany (FAU) (www.clinicaltrials.gov: NCT01318395, NCT00627952, NCT01319357, NCT02383238). Baseline data of 73 patients with T2DM (40 out of them diagnosed with hypertension), 55 healthy individuals and 134 hypertensive patients, who had SLDF measurements with good quality were analyzed. Subjects were recruited by advertising in local newspapers in the area of Erlangen-Nürnberg, Germany, and eligible subjects were enrolled consecutively. Written informed consent was obtained before study inclusion. The study protocol of each trial was approved by the Local Ethics Committee (University of Erlangen-Nürnberg), and the studies were conducted in accordance with the Declaration of Helsinki and the principles of good clinical practice guidelines. The financial sponsors did not contribute to data collection, interpretation of the data, or the decision to approve and submit the manuscript.

### Study population

Male and female Caucasian patients with mild to moderate uncomplicated essential hypertension stage 1 and 2 with mean sitting systolic BP ≥ 140 mmHg and/or diastolic BP ≥ 90 mmHg or treated arterial hypertension (www.clinicaltrials.gov: NCT01318395, NCT00627952) and patients with T2DM (defined by fasting glucose ≥ 126 mg/dl or HbA1c ≥ 6.5% or on blood glucose lowering medication) were included (www.clinicaltrials.gov: NCT01319357, NCT02383238). Patients in the diabetic group were non-insulin-requiring with no more than one oral antidiabetic medication. Diabetic patients were considered as in the early stage of T2DM, because they presented without irreversible end-organ damage and had diabetes duration of less than 10 years. Patients with fasting blood glucose > 240 mg/dl, HbA1c ≥ 10% and type 1 diabetes were excluded. Patients with eye diseases, especially cataract, diabetic and hypertensive retinopathy were excluded. Other main exclusion criteria of all 4 studies were secondary hypertension, severe hypertension (systolic BP ≥ 180 mmHg and/or diastolic BP ≥ 110 mmHg), history of hypertensive encephalopathy or intracerebral hemorrhage, history of ischemic stroke, history of epilepsy and history of coronary artery disease or heart failure within the previous six months. Patients with any other significant disease or dysfunction were excluded. Office blood pressure (BP) was taken at the same time as SLDF measurement was performed in a standardized fashion according to guidelines. As antihypertensive medication is known to influence capillary density[23] and several antidiabetic substances are known to impact blood pressure, all patients with medication prior to study enrollment underwent a four week wash-out period of either antidiabetic or antihypertensive medication before SLDF measurement was performed. The results were compared to a group of 55 healthy male and female non-smoking Caucasian individuals, who were recruited in parallel and had the same clinical work-up as patients in the clinical trials.

### Retinal arteriolar structure

To assess the vascular wall lumen diameters, SLDF at 670 nm (Heidelberg Retina Flowmeter, Heidelberg Engineering, Germany) was performed.[22] Outer arteriolar diameter (OD) was measured in reflection images, and inner (lumen) diameter (ID) was measured in perfusion images (software version SLDF 4.0). WLR was calculated using the formula (OD-ID)/ID.[24, 25] Three measurements were taken of every patient in a sitting position after 15 minutes rest. Each image was approved and validated by an investigator blinded to the clinical characteristics of patients. Data were calculated as mean of three images for each patient. No pupil dilatation was performed to prevent artificial changes in vascular tone and flow.[24] Measurements were obtained in a dark room.

### Retinal capillary flow

All measurements were performed in the juxtapapillary area of the right eye, 2 to 3 mm temporal superior of the optic nerve. For perfusion analysis, capillary vessels with diameter ≤ 20 μm were selected. Retinal capillary blood flow (RCF) was assessed using SLDF and blood flow was calculated independently by the automatic full field perfusion image analyzer (AFFPIA).[26] The mean RCF was calculated in the capillaries and intercapillary areas (area with predominance of vessels with ≤ 10 μm diameter) and expressed as arbitrary units (AU). The mean of three measurements was taken. Vessels with a diameter > 20 μm, saccades or over- and under expressed pixels were automatically detected and excluded by AFFPIA.[26] Each image was approved and validated by a scientist blinded to the clinical characteristics of patients.

### Retinal intercapillary distance

**Fig 1:**
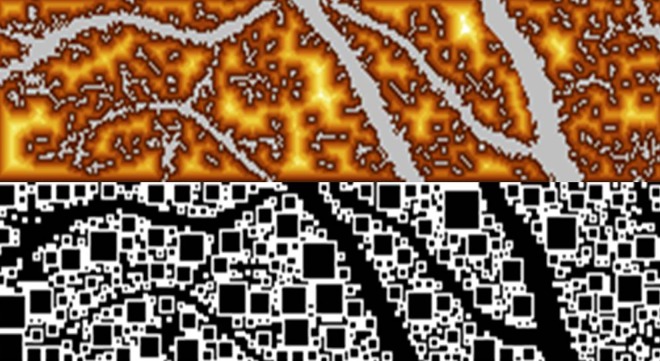
Measurement of intercapillary distance in perfusion image with scanning laser Doppler flowmetry and calculation of intercapillary distance using automatic full field perfusion image analyser

Both parameters of capillary rarefication, i.e. measurement of ICD and CapA, were determined from perfusion images of pixels. ICD was defined as distance between any pixel outside and the next pixel inside the vessel and expressed in μm (Fig 1). One pixel was defined as the smallest dot of optic solution, where flow can be detected. SLDF optic solution works in a confocal manner with pixel sizes of 10 x 10 μm. Pixels are located in a depth of 300 μm. Depending on the vessel size, pixels were categorized the following way: Pixels inside vessels > 20 μm were defined as non-capillary vessel pixels (e.g. arteriolar pixels), pixels inside vessel ≤ 20 μm were defined as capillary pixels, and pixels outside a vessel were defined as intercapillary pixels.

### Retinal capillary area

CapA was defined as area with vessels with ≤ 20 μm diameter. Thus, for calculation, area with vessel size > 20 μm was subtracted from the total area. CapA was expressed as number of pixels.

### Reliability of retinal intercapillary distance and capillary area

Assessment of reliability was performed under routine laboratory conditions with the Heidelberg Retinal Flowmetry and has been previously demonstrated [23]. Minimal biological variability was analyzed in 3 images of 5 persons. All 3 images were analyzed within 5 minutes. Coefficient of variation (CV) was 3.6 ± 3.0% for ICD and 8.53 ± 5.4% for CapA, Cronbach’s alpha revealed 0.93 for ICD and 0.87 for CapA. Maximal biological variability was assessed in 5 individuals in 3 images 3 times each within 6 weeks. CV was 9.52 ± 4.2% for ICD and 10.59 ± 2.5% for CapA, Cronbach’s alpha revealed 0.88 for ICD and 0.96 for CapA.

### Statistical methods

Primary objective was to compare rarefaction parameters in patients with T2DM and healthy individuals as well as hypertensive patients. Normal distribution of data was confirmed by Kolmogorov-Smirnov test before further analysis. Normally distributed data of all three groups was compared using univariate variance analysis ANOVA. Data showed homogeneity of variance. Therefore, Post-Hoc-Analysis was performed using Hochberg-Test. Multivariate adjustment for model 1 included age, gender, BMI, systolic office BP, HDL-cholesterol, and serum-creatinine. These parameters were chosen since they differed between the healthy control group and the diabetic group, respectively. Data is given as mean ± SD in tables. For not- normally distributed parameters, Kruskal-Wallis-Test was used for further analysis. Two-tailed values of p < 0.05 were considered statistically significant. Non-adjusted and adjusted p-values are given in the text of the result section; adjusted p-values are given in tables. Bivariate correlation analyses were performed using Pearson's test. To minimize the influence of confounding variables, partial correlation was performed with adjustment for model 1. All analyses were performed using IBM SPSS Statistics 22 (SPSS Inc, Chicago, IL/ USA).

## Results

### Study population

Baseline characteristics of the study population are given in Table 1. The diabetic study population comprised 73 patients (age 60 ± 7.3 years, 41% females), that were compared to 55 healthy individuals (age 53 ± 13 years, 66% females) and to 134 patients (age 52 ± 11 years, 26% females) with uncomplicated primary hypertension stage 1 or 2. There were significant differences in age, gender, BMI, serum creatinine, HDL-cholesterol and fasting plasma glucose between the three groups by study design (Table 1). Sixty-two out of 73 diabetic patients received antidiabetic treatment prior to study enrolment consisting of either metformin or sulfonylurea (metformin in 49% of patients with T2DM), but underwent 4 weeks of washout of antidiabetic medication prior to baseline measurements. All patients of the hypertensive group also received 4 weeks of washout of their respective antihypertensive medication, in 81% angiotensin converting enzyme inhibitors or angiotensin receptor blockers, in 24% calcium-channel-blockers, in 12% diuretics and in 6% beta-blockers.

**Table 1:** Study population.

	Diabetic patients (n = 73)	Hypertensive patients (n = 134)	Healthy individuals (n = 55)	p-value (diabetic vs. hypertensive)	p-value (diabetic vs healthy)
**Age (years)**	60 ± 7.3	52 ± 11	53 ± 13	< 0.001	0.004
**Sex (m/f)**	43/30	99/35	19/36	< 0.001	0.011
**BMI (kg/m2)**	30 ± 5.0	28.1 ± 3.6	25.0 ± 4.3	< 0.001	< 0.001
**HbA1c (%)**	6.7 ± 0.8	5.5 ± 0.3	5.6 ± 0.3	< 0.001	< 0.001
**Duration of diabetes (month)**	61 ± 50	-	-	-	
**Systolic office BP (mmHg)**	131 ± 11	146 ± 12	127 ± 18	< 0.001	0.369
**Diastolic office BP (mmHg)**	80 ± 8.9	90 ± 8.8	78 ± 9.1	< 0.001	0.669
**Systolic 24h ambulatory BP (mmHg)**	128 ± 11	145 ± 10	123 ± 12	< 0.001	0.194
**Diastolic 24h ambulatory BP (mmHg)**	78 ± 9.5	90 ± 9.7	77 ± 5.6	< 0.001	0.894
**HR (bpm)**	70 ± 9.4	72 ± 10	70 ± 13	0.217	0.992
**Duration of hypertension (month)**	126 ± 118	100 ± 102	-		
**Serum creatinine (mg/dl)**	0.80 ± 0.14	0.89 ± 0.17	0.83 ± 0.18	0.001	0.763
**Serum cholesterol (mg/dl)**	207 ± 33	219 ± 38	238 ± 46	< 0.001	< 0.001
**Seum LDL-cholesterol (mg/dl)**	142 ± 28	148 ± 35	156 ± 36	0.071	0.065
**Serum HDL-cholesterol (mg/dl)**	49 ± 10	52 ± 12	63 ± 13	< 0.001	< 0.001
**Plasma glucose (mg/dl)**	137 ± 43	91 ± 11	87 ± 17	< 0.001	< 0.001

Data are given as mean ± SD, BMI–body mass index, BP–blood pressure, LDL–low density lipids, HDL–high density lipids

### Parameters of capillary rarefaction

In diabetic patients, ICD was significantly greater than in the healthy control group (p = 0.007). After adjustment for model 1 (see methods), ICD remained significantly increased in diabetic patients compared to healthy persons (p = 0.013). There was no difference in CapA when comparing the unadjusted data (p = 0.131), but CapA was smaller in diabetic patients than in the healthy control group after adjustment for model 1 (p = 0.019) (Table 2). Thus, our data indicates that capillary rarefaction is more commonly observed in patients with diabetes compared to healthy persons.

**Table 2:** Retinal parameters.

	Diabetic patients	Hypertensive patients	Healthy subjects	p-value diabetic vs healthy	p-value diabetic vs hypertensive
**Parameters of capillary density**					
**ICD (μm)**	23.2 ± 5.5	23.1 ± 5.8	20.2 ± 4.2	0.013	0.781
**CapA (-)**	1592 ± 595	1556 ± 649	1821 ± 652	0.019	0.768
**RCF (AU)**	304 ± 75	291 ± 71	314 ± 61	0.311	0.650
**Structural parameters**					
**OD (μm)**	107 ± 11	102 ± 14	107 ± 11	0.215	0.003
**ID (μm)**	79 ± 6.8	76 ± 7.8	80 ± 8.0	0.010	0.004
**WT (μm)**	14.2 ± 3.5	13.2 ± 4.0	13.6 ± 3.8	0.553	0.027
**WLR (-)**	0.358 ± 0.09	0.345 ± 0.09	0.352 ± 0.01	0.174	0.153

Data are given as mean ± SD, adjusted p-values according to model 1 (see methods) are provided, ICD–intercapillary distance, CapA–capillary area, RCF–retinal capillary flow, OD–outer diameter of retinal arteriole, ID–inner diameter of retinal arteriole, WT–wall thickness of retinal arteriole, WLR–wall to lumen ratio.

When diabetic patients were compared to the hypertensive group, there was no difference in ICD (p = 1.0, after adjustment for model 1 p = 0.781) and CapA (p = 0.975, after adjustment for model 1 p = 0.768) thus indicating no significant difference in capillary density between individuals with T2DM and patients with hypertension.

### Structural retinal parameters

In diabetic patients, there was a difference in ID (p = 0.859, after adjustment for model 1 p = 0.010), but no difference in OD (p = 0.999, after adjustment for model 1 p = 0.215), WT (p = 0.765, after adjustment for model 1 p = 0.553) and WLR (p = 0.983, after adjustment for model 1 p = 0.174) compared to the healthy control population.

In diabetic patients compared to hypertensive patients, OD (p = 0.027, after adjustment for model 1 p = 0.003) and ID (p = 0.036, after adjustment for model 1 p = 0.004) were significantly increased. There was also a difference in WT (p = 0.199, after adjustment for model 1 p = 0.027), but not in WLR (p = 0.767, after adjustment for model 1 p = 0.153).

### Correlations

Correlation r and p-values (raw and adjusted for model 1) for all parameters described are shown in Table 3. Considering retinal capillary density and blood pressure in patients with T2DM there was no correlation between ICD as well as CapA and systolic as well as diastolic office and 24h ambulatory BP.

**Table 3:** Correlations.

Correlated parameters	Raw r and p value	r and p value after adjustment for model 1
**Capillary density and blood pressure in patients with diabetes**		
ICD and systolic office BP	r = -0.107	r = 0.167
	p = 0.366	p = 0.339
ICD and diastolic office BP	r = 0.048	r = 0.324
	p = 0.688	p = 0.058
ICD and systolic ambulatory 24h BP	r = 0.029	r = 0.077
	p = 0.858	p = 0.662
ICD and diastolic ambulatory 24h BP	r = 0.087	r = 0.219
	p = 0.592	p = 0.207
CapA and systolic office BP	r = 0.096	r = -0.091
	p = 0.417	p = 0.603
CapA and diastolic office BP	r = -0.087	r = -0.196
	p = 0.466	p = 0.259
CapA and systolic ambulatory 24h BP	r = -0.081	r = -0.171
	p = 0.617	p = 0.327
CapA and diastolic ambulatory 24h BP	r = -0.094	r = -0.232
	p = 0.564	p = 0.180
**Capillary density and parameters of diabetes control in patients with diabetes**		
ICD and diabetes duration	r = -0.034	r = 0.080
	p = 0.774	p = 0.647
ICD and HbA1c	r = 0.100	r = -0.055
	p = 0.398	p = 0.752
ICD and fasting plasma glucose	r = 0.035	r = -0.014
	p = 0.768	p = 0.935
CapA and diabetes duration	r = 0.023	r = -0.015
	p = 0.850	p = 0.923
CapA and HbA1c	r = -0.115	r = -0.039
	p = 0.332	p = 0.825
CapA and fasting plasma glucose	r = -0.069	r = -0.047
	p = 0.565	p = 0.791
**Capillary rarefaction and age in patients with diabetes**		
ICD and age	r = 0.077	r = 0.095
	p = 0.515	p = 0.439
CapA and age	r = -0.063	r = -0.058
	p = 0.597	p = 0.635
**Capillary rarefaction and age in patients with hypertension**		
ICD and age	r = -0.225	r = -0.204
	p = 0.009	p = 0.025
CapA and age	r = 0.269	r = 0.245
	p = 0.002	p = 0.007
**Capillary rarefaction and age in healthy individuals**		
ICD and age	r = -0.218	r = -0.194
	p = 0.109	p = 0.187
CapA and age	r = -0.075	r = -0.097
	p = 0.592	p = 0.511

ICD–intercapillary distance, CapA–capillary area, BP–blood pressure

When correlating retinal capillary density and parameters of diabetes control, there was no correlation between ICD as well as Cap A and diabetes duration, HbA1c and fasting plasma glucose in patients with diabetes.

In diabetic patients, there was no correlation between ICD as well as CapA and age, whereas in hypertensive patients, capillary density increased with age. In healthy individuals, there was no correlation between ICD as well as CapA and age.

### Subgroup analysis

Subsequently, diabetic patients were separated in 40 patients with and 33 patients without hypertension. Diabetic patients with hypertension showed well-controlled office and 24h BP values (Table 4). Baseline characteristics of both subpopulations are indicated in Table 4. There was no significant difference in ICD (p = 0.647) and CapA (p = 0.901) as well as in RCF (p = 0.977) OD (p = 0.546), ID (p = 0.437), WT (p = 0.846) and WLR (p 0 0.887) between diabetic patients with hypertension and well controlled BP values compared to diabetic patients without hypertension (Table 5).

**Table 4:** Subgroup characteristics of the study population with diabetes mellitus Type 2.

	Diabetes and hypertension (n = 40)	Diabetes and no hypertension (n = 33)	p-value
**Age (years)**	60 ± 8.0	60 ± 6.2	0.989
**Sex (m/f)**	21/19	22/11	0.226
**BMI (kg/m2)**	30 ± 4.5	29 ± 5.4	0.237
**HR (bpm)**	70 ± 9.9	70 ± 8.9	0.875
**Duration of diabetes (month)**	63 ± 55	59 ± 44	0.689
**Plasma glucose (mg/dl)**	137 ± 46	136 ± 40	0.960
**HbA1c (%)**	6.6 ± 0.9	6.8 ± 0.7	0.215
**Duration of hypertension (month)**	126 ± 118		
**Systolic office BP (mmHg)**	130 ± 12	132 ± 10	0.302
**Diastolic office BP (mmHg)**	78 ± 8.9	83 ± 11	0.072
**Systolic 24h ambulatory BP (mmHg)**	130 ± 12	126 ± 10	0.323
**Diastolic 24h ambulatory BP (mmHg)**	76 ± 10	80 ± 8.6	0.278
**Serum creatinine (mg/dl)**	0.80 ± 0.14	0.81 ± 0.14	0.785
**Serum cholesterol (mg/dl)**	200 ± 33	214 ± 31	0.067
**Serum LDL-cholesterol (mg/dl)**	138 ± 29	147 ± 26	0.170
**Serum HDL-cholesterol (mg/dl)**	48 ± 8.8	50 ± 12	0.464

Data are given as mean ± SD, BMI–body mass index, BP–blood pressure, LDL–low density lipids, HDL–high density lipids

**Table 5:** Retinal parameters in patients with type 2 diabetes mellitus with and without hypertension.

	Diabetes and hypertension	Diabetes and no hypertension	p-value
**ICD (μm)**	22.9 ± 5.6	23.5 ± 5.5	0.647
**CapA (-)**	1583 ± 566	1600 ± 638	0.646
**RCF (AU)**	305 ± 80	304 ± 70	0.977
**OD (μm)**	107 ± 12	106 ± 10	0.546
**ID (μm)**	78 ± 7.0	79 ± 6.7	0.437
**WT (μm)**	14.1 ± 2.9	14.3 ± 4.1	0.846
**WLR (-)**	0.359 ± 0.08	0.356 ± 0.11	0.887

Data are given as mean ± SD, ICD–intercapillary distance, CapA–capillary area, RCF–retinal capillary flow, OD–outer diameter of retinal arteriole, ID–inner diameter of retinal arteriole, WT–wall thickness of retinal arteriole, WLR–wall to lumen ratio

## Discussion

Our measurements of ICD and CapA indicate lower capillary density in patients with T2DM compared to healthy individuals, but comparable to the extent of capillary rarefaction in hypertensive patients. In the diabetic study population, there was no significant difference in capillary density in patients with hypertension (but well controlled BP) compared to patients without hypertension.

The capillary rarefaction observed in our diabetic patients is in accordance with previous findings in animal studies. Induction of diabetes in mice led to a decrease in major angiogenic growth factor in the skeletal muscle.[6] In a diabetic mouse model with induced ischemia, restoration of perfusion in the ischemic limb was impaired and capillary density reduced.[8] Under maximally dilated conditions, constant flow-perfused skeletal muscle of obese Zucker rats exhibited elevations in perfusion pressure, indicative of an increased resistance to perfusion within the microcirculation and capillary rarefaction.[27] It is questionable whether artificially induced limb ischemia in the mouse model is a good comparison for the human capillary bed. Previous investigations found that functional non-perfusion of capillary arteries as induced by limb ischemia leads to anatomical absence of capillaries on the long term, called “structural capillary rarefaction”. Whereas models of induced ischemia measure functional capillary rarefaction, our patients with T2DM for a median duration of 5 years are likely to additionally present structural absence of capillaries. Unfortunately, SLDF does not allow for distinction between structural and functional retinal capillary rarefaction. But one has to take into account that structural capillary rarefaction has also been described using histological analyses in the skeletal muscle of obese Zucker rats, an animal model of T2DM. ^[^^27^^,^ ^28^^]^

In patients with T2DM and overt end-organ damage, data about capillary density are heterogeneous. Coronary collateral vessel formation was shown to be decreased in patients with diabetes mellitus (mixed type 1 and 2) with advanced stages of coronary atherosclerosis compared with non-diabetic individuals.[3] This is in accordance with our findings of capillary rarefaction in the retinal capillary bed. Nevertheless, one has to take into account that the patients investigated in the clinical trial mentioned already suffered from coronary artery disease, whereas our study cohort did not present overt end-organ damage, which makes a direct comparison impossible. In addition, whereas the investigation of coronary collateral vessel formation requires invasive heart catheter investigation, the assessment of retinal capillary rarefaction has the advantage of being non-invasive. Two clinical trials measuring capillary density with videomicroscopy in dorsal finger skin showed no difference between healthy and glucose-intolerant subjects[10] or patients with type 2 diabetes.[14] A possible explanation might be that the diabetic patients received a blocker of the renin-angiotensin system for hypertension.[14] It has been shown that especially drugs targeting the RAS ameliorate capillary rarefaction.[29] Alternatively, it has to be questioned whether videomicroscopy in the dorsal finger skin assesses the capillary bed that is predominantly damaged by diabetes. For that reason, we chose the retinal circulation (but in patients without diabetic retinopathy), a clear target of diabetes that moreover may mirror the cerebrovascular circulation.[18–20] In cerebral capillaries, rarefaction has so far been shown in animal models only, but findings are consistent with our results observed in the retinal circulation in-vivo in humans that mirrors the cerebrovascular circulation. [16], [30]

In our study cohort of diabetic patients with median diabetes duration of 60 months, we found no difference in WLR and WT in patients with T2DM compared to healthy controls and hypertensive individuals. This is in accordance with previous data from our study group showing that only patients with long-term T2DM (diabetes duration of >60 month) presented retinal arteriolar hypertrophic remodeling with increased WT compared to healthy individuals and hypertensive patients.[31] Previous data from gluteal arteriolar biopsies indicated hypertrophic arteriolar remodeling, but as previously discussed by our group[31], in these patients no adjustment has been made for potentially important confounding factors such as e.g. age. Our results are in accordance with previous data from coronary arterioles.[32] Of note, patient groups in the analysis of coronary arterioles did not differ significantly in age. Overall, the known confounding impact of age and diabetes duration on structural arteriolar changes resulted in the investigation of short term functional vessel changes, such as retinal capillary rarefaction in this analysis.

In our study population, there was no difference in RCF between the groups. This is in accordance with two clinical studies also indicating no difference in basal RCF in normotensive and hypertensive patients stage 1 or 2.[33, 34] Nevertheless, Ritt and colleagues described an impaired increase of retinal capillary blood flow to flicker light exposure in arterial hypertension.[35] A possible explanation for the differing results might be that RCF has been shown to be negatively related to WLR in hypertensive patients, independently of cardiovascular risk factors[35] and no changes were now found in WLR, also reflecting the fact that our patients were in the early stage of their desease.

In our hypertensive group, capillary density increased with age. There were no associations between capillary density and age in the diabetic group and the healthy control group. Fenton found that conjunctival microvessels per unit area of conjunctival tissue increased progressively with age in diabetic and non-diabetic individuals.[11] The increase with age was found to be more pronounced in non-diabetics compared to diabetics because diabetic patients had substantially fewer small vessels compared to nondiabetics.[11] This is in accordance with our findings of reduced capillary density in diabetic patients compared to non-diabetic patients and might also explain why no association between age and capillary density has been observed in our study cohort with T2DM.

Duration of diabetes and HbA1c values are known determinants of end-organ damage in diabetic patients. We demonstrated for the first time retinal capillary rarefaction in diabetic patients with mean disease duration of 5 years (mean HbA1c 6.7%). Skin capillary rarefaction has been previously described in patients with mean diabetes duration of 10–11 years.[11] Our finding of capillary rarefaction in the early stage of type 2 diabetes mellitus is in accordance with data describing a limited vasodilator capacity in skin vessels in some patients at the time of diagnosis[36] and also in prediabetic subjects[37] with impaired glucose tolerance.[10]

In hypertensive patients. capillary density has been previously shown to inversely correlate with office BP levels in capillaries of the skin[38–40] and cerebral capillaries.[41] Even though half of our diabetic study population had previously diagnosed hypertension, there was no difference in capillary rarefaction between patients with diabetes and hypertension and patients with diabetes and without hypertension. This finding can be explained by the well-controlled BP in our diabetic patients.

According to the current standard of care, screening for diabetic retinopathy in patients with T2DM is recommended once yearly.[42] In future, measurement of retinal capillary rarefaction with SLDF might be included in the yearly assessment. We suggest that the assessment of capillary rarefaction might be superior to retinopathy screening to identify patients at risk of developing end-organ damage at a very early stage. Diabetic patients presenting with retinopathy already show potentially non-reversible severe microvascular damage. On the contrary, retinal capillary rarefaction has the advantage to be reversible after antihypertensive therapy and therefore might present an interesting target for further investigation.

### Limitations

This is a comparative study of patients, who participated in four large clinical trials from 2005–2015 in our Clinical Research Unit. Baseline characteristics of the study population including cardiovascular risk factors differed between the three study populations. Therefore, multivariate adjustment was performed to minimize the confounding effects, and all measurements of capillary density were evaluated by the same person and according to the same protocol.

Our study population was selected from a larger group according to quality of SLDF measurements, which was necessary to identify ICD and CapA precisely. Nevertheless, we are convinced that this did not affect the observed associations between hypertensive status and parameters of capillary rarefaction, since all hypertensive patients showed similar values of duration and severity of diabetes and hypertension, lack of overt end-organ damage to those with valid evaluation of ICD and CapA.

Some persons of our healthy study cohort might have been pre-diabetic based on the median HbA1c and standard deviation of our healthy population (5.6 ± 0.3%), which is a clear limitation of our analysis.

Most patients of the diabetic and the hypertensive group received previous antidiabetic and antihypertensive treatment, respectively, which might have influenced our baseline-measurements of capillary density. But those patients with diabetes or hypertension previously treated received a 4 week wash-out period of the diabetic and antihypertensive medication, respectively.

It has been demonstrated in skin capillaries that rarefaction occurs due to a reduction of the number of spontaneously perfused capillaries or/ and to a decreased total number of capillaries [38]. Our measurements are based on perfusion images and therefore mirror predominantly functional capillary changes. Clearly, our method does not allow differentiating between functional and structural vessel changes.

## Conclusion

The assessment of retinal capillary density with SLDF allows for a non-invasive insight into the microcirculation. We investigated capillary rarefaction at an early stage of type 2 diabetes mellitus in patients without overt end-organ damage and found reduced capillary density compared with healthy control subjects. Our findings therefore extend the previous data about capillary rarefication in patients with overt end-organ damage. Changes in capillary density in patients at the early stage of type 2 diabetes mellitus might help to classify patients at risk of developing end-organ damage in the future.

## Abbreviations

AU: Arbitrary units
BMI: Body mass index
BP: Blood pressure
CA: Cross sectional area
CapA: Capillary area
HDL: High density lipids
ICD: Intercapillary distance
ID: Inner vessel diameter
LDL: Low density lipids
OD: Outer vessel diameter
RCF: Retinal capillary flow
SLDF: Scanning laser Doppler flowmetry
T2DM: Type 2 diabetes mellitus
WLR: Wall to lumen ratio
WT: Wall thickness
